# The Study on Newly Developed McAb NJ001 Specific to Non-Small Cell Lung Cancer and Its Biological Characteristics

**DOI:** 10.1371/journal.pone.0033009

**Published:** 2012-03-30

**Authors:** Shiyang Pan, Fang Wang, Peijun Huang, Ting Xu, Lixia Zhang, Jian Xu, Qing Li, Wenying Xia, Ruihong Sun, Lei Huang, Ying Peng, Xuejun Qin, Yongqian Shu, Zhibin Hu, Hongbing Shen

**Affiliations:** 1 Department of Laboratory Medicine, the First Affiliated Hospital of Nanjing Medical University, Nanjing, China; 2 National Key Clinical Department of Laboratory Medicine, Nanjing, China; 3 Department of Oncology, the First Affiliated Hospital of Nanjing Medical University, Nanjing, China; 4 Department of Epidemiology and Biostatistics, Cancer Center of Nanjing Medical University, Nanjing, China; Cincinnati Children's Hospital Medical Center, United States of America

## Abstract

Monoclonal antibody (McAb) is the key tool for cancer immunodiagnosis and immunotherapy. McAb-based immunotherapy that targets tumor antigens has had great achivement. In this study, a cell clone which kept secreting high-titer IgG1-type McAb named NJ001 against human non-small cell lung cancer (NSCLC) cells was obtained. The titer of purified NJ001 was 2×10^6^. The antigen named SP70 of NSCLC specifically identified by NJ001 was proved to be a protein with the relative molecular mass (Mr) of 70 kDa. The results of immunohistochemical staining indicated that NJ001 could positively react to NSCLC, but weak positively or negatively react to human small-cell lung cancer (SCLC), pulmonary pseudotumor and other epithelial tumors. In soft agar assay, the colony formation efficiency in NJ001 groups decreased in a dose-dependent manner. For the concentration of 100 µg/ml, 200 µg/ml and 400 µg/ml, the inhibition ratio of colony formation was 23.4%, 62.5% and 100% respectively. Meanwhile, NJ001 caused significant reduction in tumor volume and tumor weight compared to control mice in lung cancer xenograft model. The tumor growth inhibition ratio in 200 µg, 400 µg and 800 µg NJ001 groups was 10.44%, 37.29% and 44.04%, respectively. NJ001 also led to cytomorphological changes and induced the apoptosis of human lung adenocarcinoma cell line SPC-A1 significantly. The newly developed NJ001 selectively reacted to NSCLC and exhibited anti-tumor activity both *in vitro* and *in vivo*. NJ001 is of great value concerning immunodiagnostics and immunotherapy for NSCLC and holds promise for further research regarding the mechanism underlying tumor progression of NSCLC.

## Introduction

Lung cancer is one of the most prevalent cancers and is the leading cause of cancer death due to the lack of a validated or effective screening approach for early detection. This public health burden is evident worldwide with 1.5 million lung cancer related deaths in 2010 [1]. Non-small cell lung cancer (NSCLC) accounts for more than 85% of lung cancer and most patients with NSCLC have advanced disease at diagnosis. The five-year survival rate for breast, colon, and prostate cancer, in which screening tests are available, is four to six times longer than lung cancer. The high morbidity/mortality and failure to achieve an early diagnosis result in the dismal prognosis [2], [3].

The therapies for lung cancer are primarily based on traditional modes such as surgical resection, chemotherapy, and radiotherapy; however, the curative effect obtained is less than satisfactory [4]–[6]. Recently, immunotherapy for cancer has become a method utilized as a follow-up to traditional therapy. Antibodies are becoming a major drug modality due to their high specificity and affinity to targets. Over two dozen therapeutic monoclonal antibodies (McAbs) are currently approved for the treatment of cancer and other human diseases [7]–[9]. Identification of novel antigens will further improve tumor immunotherapy. Antibody-based immunotherapy that targets tumor antigens or cell surface markers has achieved some success as a cancer therapy, including in NSCLC, with agents such as cetuximab, panitumumab, matuzumab, and trastuzumab [10]–[14].

In the present study, we produced a monoclonal antibody designated NJ001, generated by immunizing mice with human SPC-A1 lung adencarcinoma live cell antigen. The anti-tumor activity of McAb NJ001 was exhibited both *in vitro* and i*n vivo*.

## Results

### Production and Characterization of NJ001

**Figure 1:**
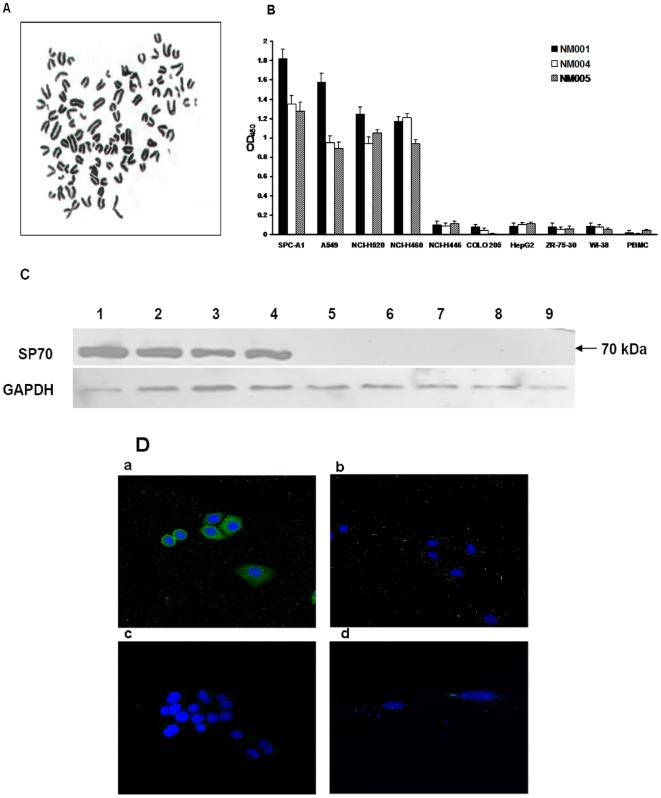
Production and characterization of NJ001. (A) Karyotype of NM001 hybridoma cell line (×400). (B) Binding activity of McAbs to human maligant and nonmaligant cells in culture. Undiluted supernatants from hybridomas NM001, NM004, NM005 were tested in triplicate with indirect cell ELISA as described in “Materials and Methods”. Each number represents the average absorbance of the substrate end product at 450 nm. Controls without McAbs exhibited an average absorbance of 0.02. NM001 was selected for further study as it exhibited the greatest binding ratio with lung tumor cells and the greatest specificity for the lung cancer cell lines compared with the other tumor cell lines. (C) Western blot analysis for the reaction of NJ001 produced from hybridoma NM001 with different cells (human maligant and nonmaligant cells) in culture. (Lane 1, SPC-A1; lane 2, A549; lane 3, NCI-H520; lane 4, NCI-H460; lane 5, HepG2; lane 6, ZR-75-30; lane 7, COLO 205; lane 8, WI-38; lane 9, PBMC; lane 10, Marker). GAPDH was used as a loading control. Results indicated that the expression of protein was specific to antibody NJ001 in non-small cell lung cancer cell lines, not in other cancer cell lines and normal cells. (D) Representative positive and negative results obtained from IIF analysis of reaction of NJ001 with different cells observed by fluorescence and confocal microscopy analysis (×400). (a, SPC-A1; b, ZR-75-30; c, HepG2; d, WI-38). Presence of NJ001 can be visualized in cellular cytoplasm of SPC-A1 cells, but other cell lines showed no fluorescence. Localization of the antigen specific to NJ001 may be in cellular cytoplasm of SPC-A1 cells.

Upon immunizing BALB/c mice with human lung adenocarcinoma cells, 3 positive monoclonal hybridoma cell lines (NM001, NM004, NM005) were confirmed by repeated indirect cell ELISA testing to continuously produce antibodies and were thus selected for expansion and recloning. Chromosome numbers of each hybridoma karyotype were more than 95. Figure 1A was the karyotype of NM001 hybridoma cell. Purified McAbs from ascites were acquired by Protein A affinity purification. The McAb from NM001 had the highest titer among the three antibodies, reaching 2×10^6^. NM001 and NM004 kept secreting IgG1, κ- type McAb, while NM005 secreted IgG2b, κ- type McAb.

Each McAb was characterized by the results of binding to a panel of normal and malignant cells listed in Figure 1B. By ELISA analysis, we determined that McAbs produced by the hybridoma cell lines NM001, NM004 and NM005 exhibited higher binding ratios with the NSCLC cell lines SPC-A1, A549, NCI-520 and NCI-H460, but exhibited generally lower binding ratios in SCLC cell line NCI-H446, other cancer and normal cell lines. NJ001, produced by the hybridoma cell line NM001, was selected for further study because it exhibited the highest specificity for the lung cancer cell lines compared to other tumor cell lines.

Western blot analysis results were consistent (Figure 1C). We could only detect the expression of the protein named SP70 specific to NJ001 in NSCLC cell lines, but not in other cancer cell lines and normal cells.

Indirect immunofluorescence results were shown in Figure 1D. SP70, recognized by NJ001, was localized in the cell memberane and cellular cytoplasm of SPC-A1 cells, whereas the other cell lines exhibited no fluorescence.

### Immunohistochemical Analysis

**Figure 2:**
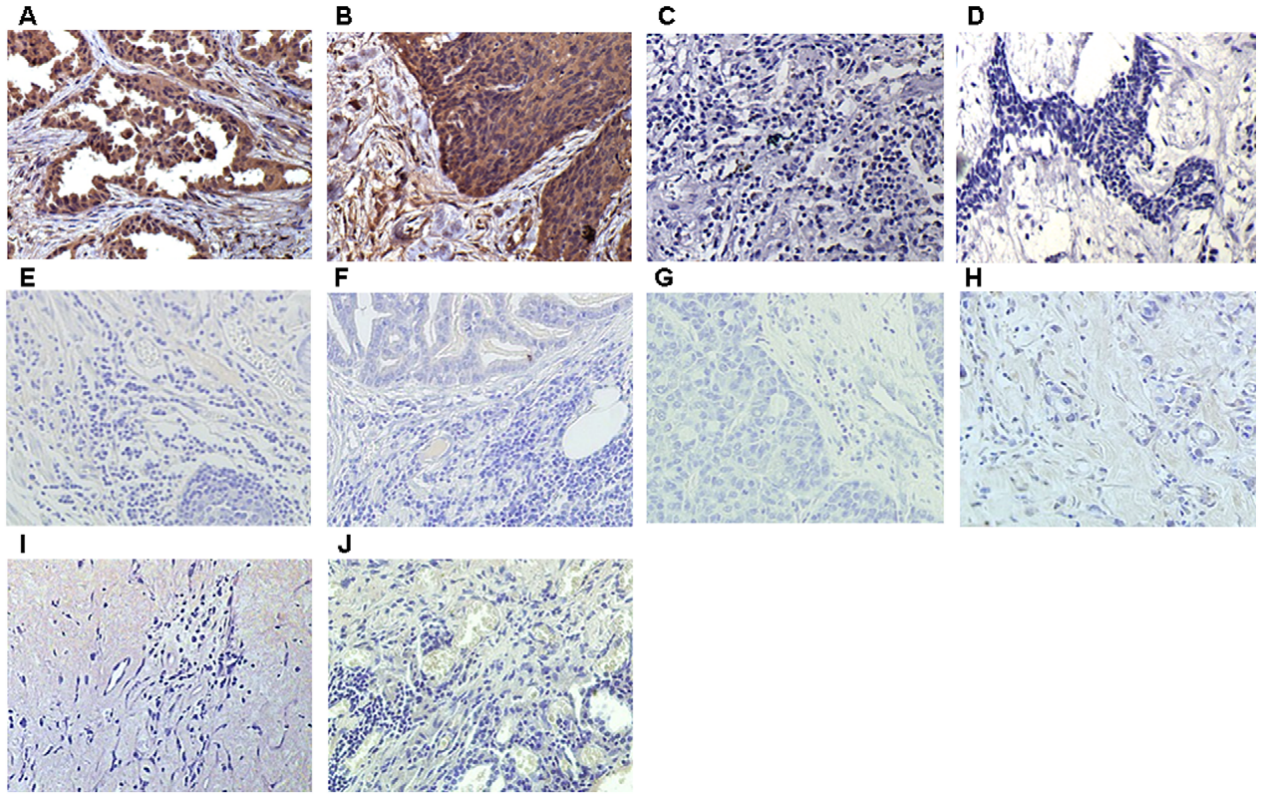
Photomicrographs of immunohistochemistry staining with NJ001 (×200). Representative areas of tumor sections from (A) NSCLC lung adenocarcinoma; (B) NSCLC squamous lung cancer; (C) SCLC; (D) Breast carcinoma; (E) Gastric cancer; (F) Colon cancer; (G) Ovarian cancer; (H) Liver cancer; (I) Pulmonary pseudotumor; (J) Adjacent nontumourous lung tissues.

Immunohistochemical analysis results showed that the expression of the SP70 was strong positive in lung adenocarcinoma tissue and squamous lung cancer tissue (Figure 2A, 2B), while weak positive or negative expression was observed in the tissue of SCLC, breast carcinoma, gastric cancer, colon cancer, ovarian cancer and liver cancer (Figure 2C, 2D, 2E, 2F, 2G, 2H). SP70 was not found in the tissues of pulmonary pseudotumor and adjacent nontumourous lung tissues (Figure 2I, 2J).

SP70 expressions in the tissue of lung adenocarcinoma, squamous lung cancer, SCLC, breast carcinoma, gastric cancer, colon cancer, ovarian cancer, liver cancer, pulmonary pseudotumor and adjacent nontumourous lung were 58/58, 48/48, 2/21, 3/21, 1/5, 0/5, 0/5, 1/5, 0/25 and 0/8 respectively (Table 1).

**Table 1:** Analysis the reactivity of NJ001 in different tissues by immunohistochemistry staining.

Pathological types	Number of samples	Number of samples positive reaction to NJ001	Integrated value
NSCLC (Lung adenocarcinoma)	58	58	+++
NSCLC (Squamous lung cancer)	48	48	++∼+++
SCLC	21	2	+
Breast carcinoma	21	3	+
Gastric Cancer	5	1	+
Colon Cancer	5	0	−
Ovarian cancer	5	0	−
Liver Cancer	5	1	+
Pulmonary pseudotumor	25	0	−
Adjacent nontumourous lung tissue	8	0	−

SP70 was scored by multiplication of the percentage of positive tumor cells and staining intensity according to other studies. Initially, the percentage of positive cells was scored as: 1(<1/3), 2(1/3–2/3), and 3(>2/3). Thereafter, intensity of staining was graded as follows: 0 = not detected, 1 = weak, 2 = moderate and 3 = strong. The final integrated value of frequency and intensity was derived with the formula: Integrated value = A×B, A and B are scores of percentage of positive cells and intensity of staining respectively, according to the criteria mentioned above. Results were recorded as: −(A×B = 0), +(A×B = 1–2), ++(A×B = 3–4), +++(A×B = 6–9). All the immunohistochemistry results were assessed by 3 independent investigators who were blinded to clinical data. When a discrepancy was found, a consensus was reached using simultaneous examination by all the 3 investigators.

**Table 2:** Results of colony formation of SPC-A1 cells treated by NJ001 or MCA2849 in soft agar.

		NJ001				MCA2849	
concentration of NJ001(µg/mL)	Average number of colonies(≥50 cells/colony)	colony formation efficiency^a^ (%)	inhibition ratio of colony^b^ (%)	concentration of MCA2849(µg/mL)	Average number of colonies(≥50 cells/colony)	colony formation efficiency^a^ (%)	inhibition ratio of colony b (%)
0	192±7.07	0.96	0	0	202±9.71	1.01	0
100	147±12.73	0.735	23.4	100	191±7.63	0.96	5.45
200	72±4.24	0.36	62.5	200	182±6.08	0.91	9.90
400	0	0	100	400	183±5.77	0.92	9.41
800	0	0	100	800	186±10.0	0.93	7.92
1000	0	0	100	1000	183±11.26	0.92	9.41

a , colony formation efficiency = (average number of colonies/average number of cells added per well)×100%.

b , inhibition ratio of colony formation = (1- average number of colonies in NJ001 or MCA2849 group/average number of colonies in McAb free group)×100%.

### Inhibitory Effects of NJ001 on SPC-A1 Proliferation and Colony Formation

**Figure 3:**
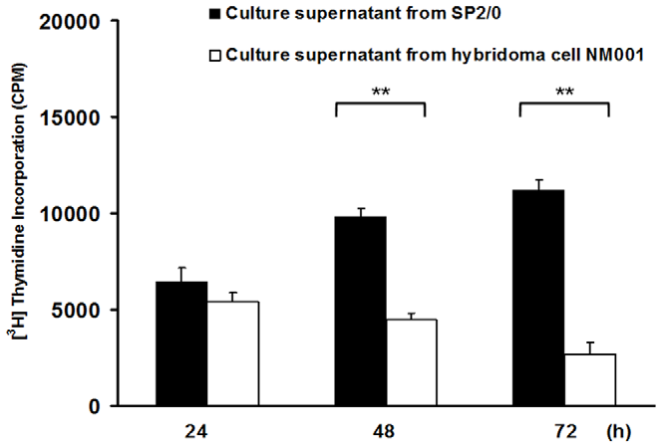
Inhibitory effect of NJ001 on SPC-A1 cell proliferation. Compared with the control treated cells, the level of proliferation of NJ001 treated SPC-A1 cells significantly decreased after 48 h and 72 h (** *P*<0.001).

The effect of NJ001 on the proliferation of SPC-A1 cells was evaluated via a [^3^H] thymidine proliferation assay. Compared with the control treated cells, the proliferation level of NJ001 treated SPC-A1 cells significantly decreased after 48 h and 72 h (*P*<0.001, *P*<0.001) (Figure 3).

**Figure 4:**
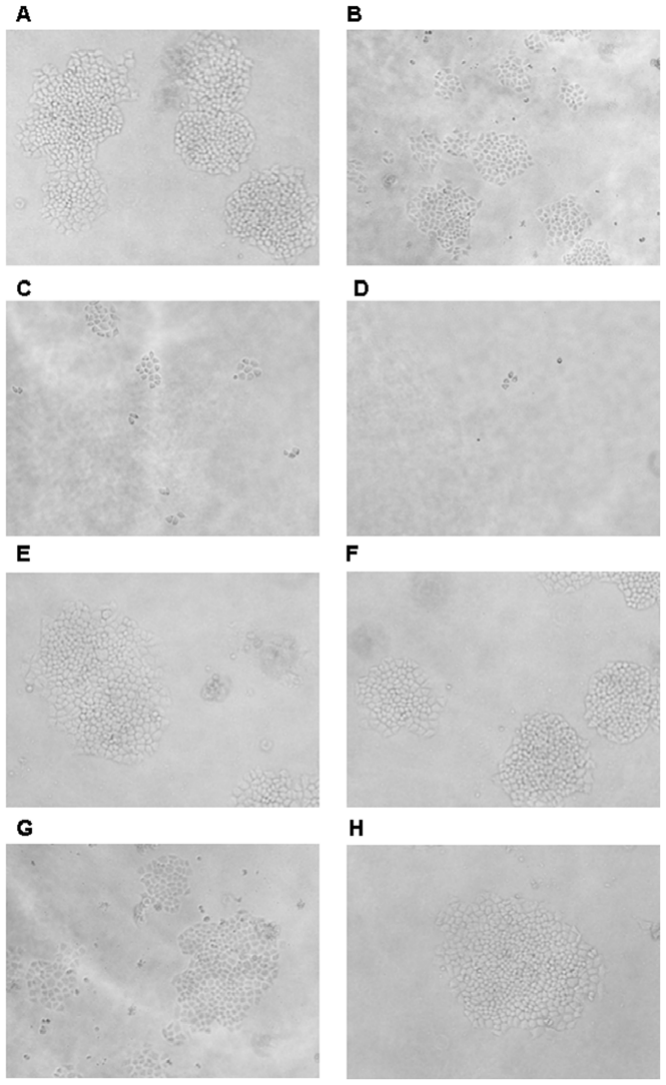
Inhibition of colony formation of SPC-A1 cells by NJ001 in soft agar. (×100). The cell suspensions (2×10^4^ cells) mixed with 0.3% agarose and different concentrations of NJ001 or MCA2849 were layered on the top of culture media in 6-well culture plates and allowed to grow for 2 weeks before colonies were counted. Representative contrast images were shown. (A) 0 µg/mL NJ001, (B) 100 µg/mL NJ001, (C) 200 µg/mL NJ001, (D) 400 µg/mL NJ001, (E) 0 µg/mL MCA2849, (F) 100 µg/mL MCA2849, (G) 200 µg/mL MCA2849, (H) 400 µg/mL MCA2849.

SPC-A1 cells were plated on a soft agar matrix, treated with NJ001 or MCA2849 (irrelevant McAb) (0, 100, 200, 400, 800, or 1000 µg/mL) and incubated at the condition of 37°C with 5% CO_2_. After 14 days, the number of colonies was counted and the representative images were obtained (Figure 4). As shown in Table 2, NJ001 inhibited colony formation in a dose-dependent manner, exhibiting 23.4% inhibition ratio at 100 µg/mL, 62.5% inhibition ratio at 200 µg/mL. When the concentration reached 400 µg/mL or higher, there were no colonies larger than 50 cells, showing a 100% inhibition ratio. However, MCA2849 didn't inhibit the colony formation of SPC-A1.

These results suggested that NJ001 effectively inhibited SPC-A1 cell proliferation *in vitro*.

### Inhibitory Effects of NJ001 in the Human SPC-A1 Lung Adenocarcinoma Mouse Xenograft Model

**Figure 5:**
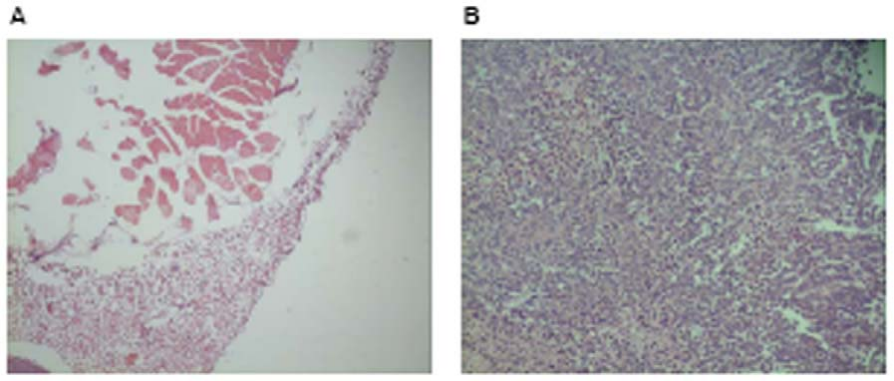
Photomicrographs of H&E staining in the preliminary study (×200). Representative areas of tissue sections from inoculation sites in NJ001 group (A) and the excised tumors in control group (B).

In the preliminary study, after 3 weeks of inoculation, the mice were euthanized. The tissues from the injection site in the incubation group and the tumors in the control group were excised. Histopathology showed no tumor growth in tissues of the incubation group and tumor growth in tissues of the control group (Figure 5).

**Figure 6:**
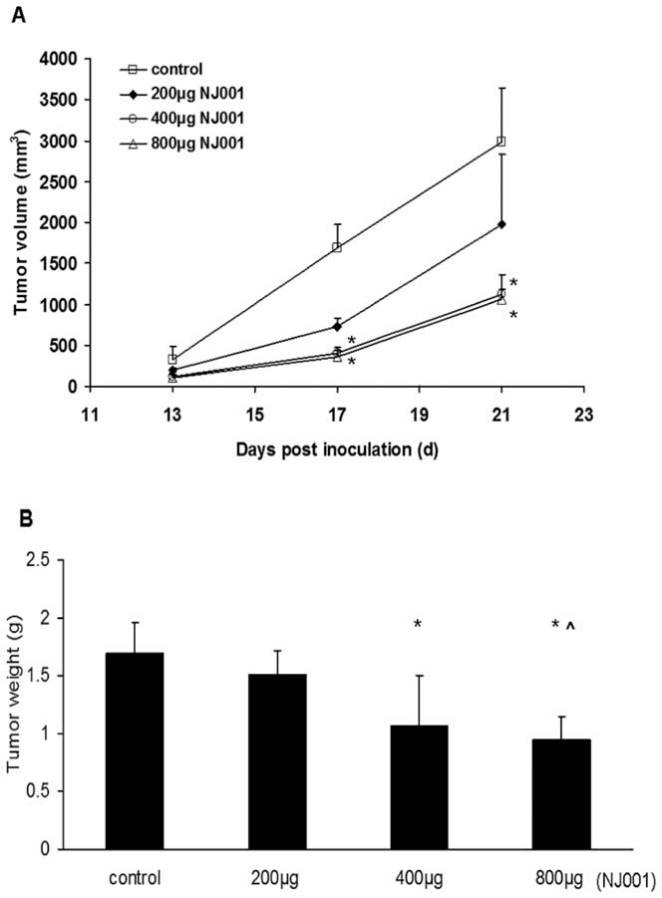
Inhibition of tumor growth *in vivo* by NJ001 in the SPC-A1 xenograft model. (A) Tumor growth curve. Animals were subcutaneously injected with 2×10^6^ SPC-A1 cells and intraperitoneally injected with normal saline, 200 µg, 400 µg, or 800 µg NJ001. Tumor volumes were measured at 4-day intervals. The error bars represent standard deviation. (B) Average tumor weight in the antibody and control groups. After 3 weeks of treatment, tumors were excised and weighed. The error bars represent standard deviation. * *P*<0.05 compared to the control group. **∧** *P*<0.05 compared to the 200 µg NJ001 group.

The result of *in vivo* experiment was shown in Figure 6A. The administration of NJ001 caused varying degrees of reduction in tumor volume compared with the saline-treated control mice. The tumor volumes in the 400 µg and 800 µg NJ001 group were significantly smaller compared to the control group 17 days after inoculation; moreover, the difference persisted to the end of the treatment (*P* = 0.004, *P* = 0.003). After 3 weeks of treatment, tumors were excised and weighed. In 200 µg, 400 µg and 800 µg NJ001 groups, tumor growth inhibition ratio [(C-T)/C%] was 10.44%, 37.29%, and 44.04%, respectively. The inhibition ratio in 400 µg and 800 µg NJ001 group was statistically significant compared to the control group (Figure 6B, *P* = 0.032, *P* = 0.015). At the end of 3 weeks, the average tumor weight in the 200 µg and 800 µg NJ001 group was (1.51±0.20) g and (0.94±0.19) g, and the difference between the two treatments was also statistically significant (*P* = 0.048).

### Apoptosis of SPC-A1 Cells Induced by NJ001

**Figure 7:**
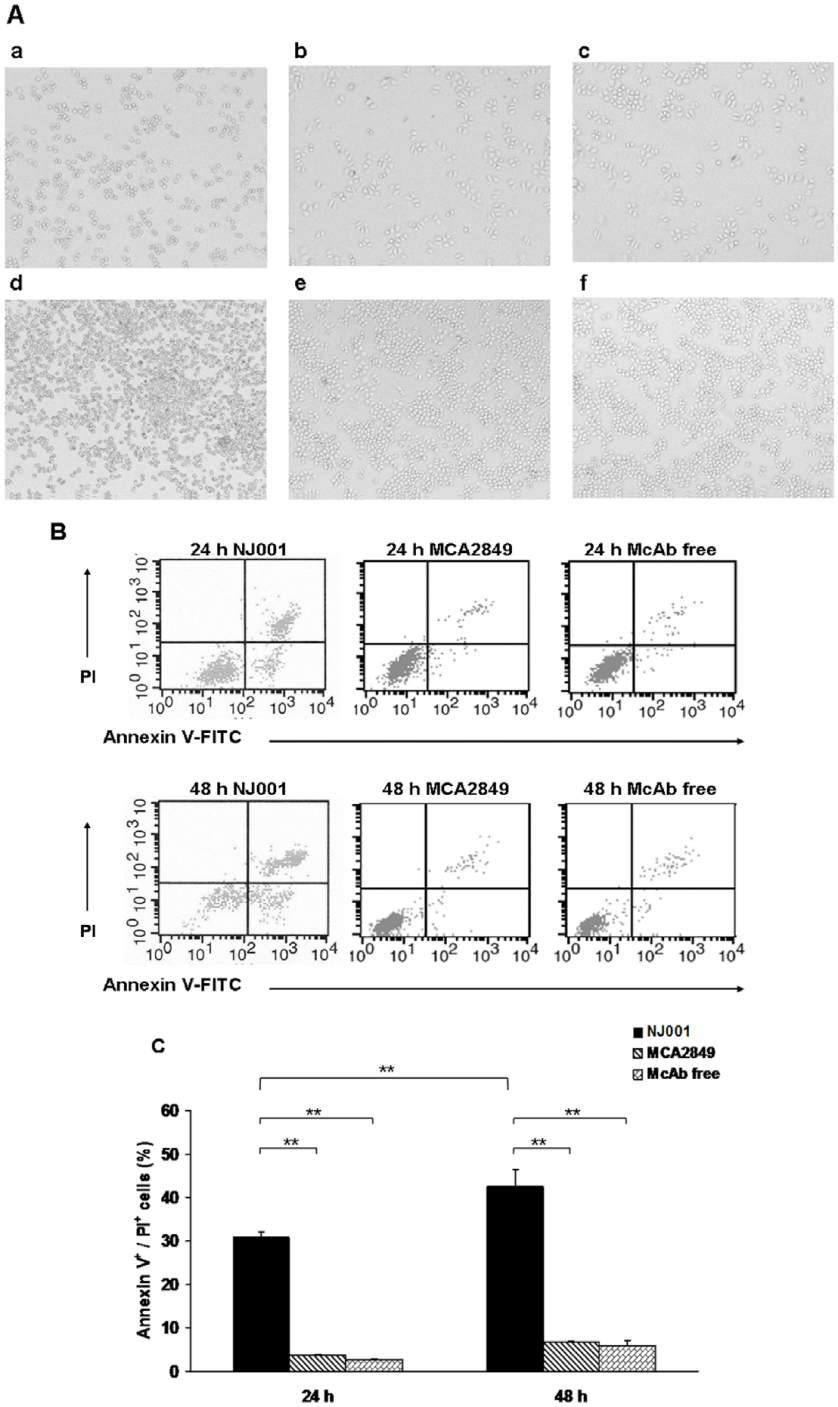
NJ001 induced apoptosis of SPC-A1 cells. SPC-A1 cells were cultured with or without 200 µg/mL NJ001 or MCA2849 for 24 h and 48 h. (A) Morphological changes in SPC-A1 cells were observed under inverted microscope (×100). a, 24 h NJ001; b, 24 h MCA2849; c, 24 h McAb free; d, 48 h NJ001; e, 48 h MCA2849; f, 48 h McAb free. (B) Apoptosis was analyzed by flow cytometry. (C) Each column and error bar represents the mean ± SD of three independent experiments (**P<0.001). The amount of late apoptosis was determined as the percentage of Annexin V^+^/PI^+^ cells.

As shown in Figure 7A, SPC-A1 cells in the NJ001 group exhibited a marginalized and condensed chromatin matrix, as well as shrinkage and blebbing of the cytoplasm and fragmented nuclei, which are typical features of apoptosis. In contrast, cells in either MCA2849 group or McAb free group maintained a normal morphology and retained an adequate ability to proliferate.

Compared to MCA2849 group and McAb free group, the high percentages of Annexin V^+^ cells (total apoptotic rate) in NJ001 group were observed at the time point of 24 h and 48 h (*P*<0.001 for both time points). The difference of total apoptotic rate in NJ001 group between 24 h time point and 48 h time point was also statistically significant (*P* = 0.002, Figure 7B).

As shown in Figure 7C, the percentages of Annexin V^+^/PI^+^ cells (late apoptotic rate) in NJ001 group, MCA2849 group and McAb free group were 30.89%, 2.80% and 3.58% at 24 h time point respectively. The late apoptotic rate in NJ001 group was also higher than the other two groups at 48 h time point (*P*<0.001, *P*<0.001). Moreover, the late apoptotic rate in NJ001 group significantly increased after 24 h (from 24 h to 48 h time point) (*P*<0.001).

NJ001 induced the apoptosis of SPC-A1 cells in a time–dependant manner.

## Discussion

Hybridoma technology is an available tool that potentially can produce anti-tumor antibodies and identify novel tumor antigens. In the past several years, considerable progress has been made in the identification of tumor-associated antigens recognized by McAbs or autoantibodies from patients. Currently, over 1,000 tumor-associated antigens have been reported [15]–[20].

In the present study, 3 McAbs were produced from 3 positive monoclonal hybridoma cell lines (NM001, NM004, NM005) that reacted in varying degrees to lung cancer cells, normal cells, and the other cancer cell lines. McAb NJ001 was selected for further study as it exhibited the highest binding ratio with lung cancer cells and also exhibited the greatest specificity for the lung cancer cell lines as compared with the other cancer cell lines. The results of immunohistochemical staining indicated that NJ001 could positively react to NSCLC, but weak positively or negatively react to human small-cell lung cancer (SCLC), pulmonary pseudotumor and other epithelial tumors.

In addition to the high affinity and specificity, NJ001 also exhibited anti-tumor activity both *in vitro* and *in vivo*. We observed the effect of NJ001 on the proliferation of lung adenocarcinoma cell line SPC-A1. Results of soft agar assay showed that the colony formation efficiency in NJ001 groups reduced in a dose-dependent manner. The xenograft was established by subcutaneous injection of SPC-A1 cells and NJ001 was administered intraperitoneally at 3 different doses. NJ001 caused varying degrees of decrease in tumor volume and tumor weight compared with control mice. Moreover, when we injected the same amount of SPC-A1 cells cultured with NJ001 for 2 h in the same way, there was no tumor growth in nude mice. We also found that NJ001 induced the cytomorphological changes and significantly induced the apoptosis of SPC-A1 cells in a time-dependent mode. This suggested that the cell apoptosis induced by NJ001 is potentially the mechanism of the anti-tumor activity. Induction of apoptosis is mediated either through death receptors (an extrinsic pathway), or at the mitochondrial level (an intrinsic pathway) [21]–[23]. Further identification of the apoptotic signaling pathways in SPC-A1 cells treated with NJ001 would be helpful in elucidating the mechanisms by which NJ001 cause anti-tumor activity both *in vitro* and *in vivo*. Whereas, functional assays in our study were performed only on SPC-A1 cells used to generate NJ001. In the next study, we will do more work to observe the growth inhibitory effects of NJ001 extended beyond a single cell line and make it clear whether the biologic activity is specific to the cell line tested or represents a more generalized NSCLC response.

The importance of tumor antigens lies in their diagnostic and potential therapeutic utility [24]–[33]. Additionally, tumor antigens can also provide prognostic information for the cancer patients [34]. The tumor-associated antigens of human lung cancer have been recognized for many years; however, few reports have investigated the common antigens or common epitopes of lung cancer [35], [36]. In this study, the antigen which was finally named SP70 recognized by NJ001 was proven to be a protein with a Mr of 70 kDa. Visualization of NJ001 binding by indirect immunofluorescence indicated that SP70 was located in cytoplasm of SPC-A1. SP70 is a potential biomarker and therapeutic target for the immunotherapy of NSCLC.

In order to explore the function of NJ001 and the corresponding Ag, more work is needed to evaluate the clinical applicability. Furthermore, the marriage of target identification with antibody enhancement technologies will ultimately be translated into new and improved therapies for cancer patients, thus providing further support as to the importance of the continued study of NJ001 [7], [37]–[39].

## Materials and Methods

### Ethics Statement

This study was carried out in strict accordance with the recommendations in the guide for the Care and Use of Laboratory Animals of the National Institutes of Health. The protocol was approved by the Committee on the Ethics of Animal Experiment of the First Affiliated Hospital of Nanjing Medical University (Permit Number: 19A5-2373). All efforts were made to minimize suffering.

Mononuclear cells (PBMC) from heparinized peripheral blood were recovered from healthy adult donors of the first affiliated hospital of Nanjing Medical University. For immunohistochemistry assay, NSCLC tissues (n = 106), SCLC tissues (n = 21), breast carcinoma tissues (n = 21), gastric cancer tissues (n = 5), colon cancer tissues (n = 5), ovarian cancer tissues (n = 5), liver cancer tissues (n = 5), pulmonary pseudotumor tissues (n = 25) and adjacent nontumourous lung tissues (n = 8) were obtained from the department of pathology in the same hospital between July 2009 and June 2010.

This study was approved by the Committee on the Ethics of Treatment of Human Subjects of the First Affiliated Hospital of Nanjing Medical University, and a written informed consent was also obtained from each participant.

### Cells and Cell Lines

Ten different human cell lines or cultures (listed in Figure 1B) representing various normal and neoplastic tissues were used to characterize the antibodies in this study. All of the cell lines were purchased from cell bank of the Chinese Academy of Sciences in Shanghai. Human lung cancer cell lines SPC-A1, NCI-H520, NCI-H460, and NCI-H446, colon carcinoma cell line COLO 205 and normal fetal lung cell line WI-38 were grown in RPMI1640 medium supplemented with 10% (v/v) fetal bovine serum (FBS) (Gibco Invitrogen, Carlsbad, CA). Human lung cancer cell lines A549, breast carcinoma cell line ZR-75-30, liver carcinoma cell line HepG2 were cultured in RPMI1640 supplemented with 10% (v/v) FBS. Mononuclear cells (PBMC) from heparinized peripheral blood were recovered from healthy adult donors (from the first affiliated hospital of Nanjing Medical University) by Ficoll-Hypaque density gradient centrifugation.

### Generation of McAb

Monoclonal antibodies were produced as follows. 6–8 week old female BALB/c mice were intraperitoneally immunized with 1×10^6^ SPC-A1 cells three times over a 3–4 week interval, then spleen cells were hybridized with SP2/0 (BALB/c mice myeloma cell line) in 50% PEG-4000 (Sigma, USA) grown in HAT medium in RPMI1640 (GIBCO, USA). Culture supernatants were screened for antibody reactivity to WI-38 and SPC-A1 cells using a live cell, solid- phase ELISA. Target cells (1×10^5^) were first plated in 96 wells and incubated for 18 h to 24 h at 37°C in 5% CO_2_. The growth medium was then aspirated and the cells were fixed for 15 min at room temperature with 95% ethanol. The cell membranes were broken in triton X-100 for 20 min and the cells were then blocked with 5% BSA for 60 min at room temperature.

The positive hybridoma cells were subcloned using a limiting dilution. Monoclonal hybridoma cells with a high valence against SPC-A1 cells were expanded and retransfused into the abdominal cavity of the BALB/c mice to prepare the ascites. The McAbs were further purified from the ascites via Protein A affinity chromatography.

The positive hybridoma cell were treated with colchicine. After 10% Giemsa dyeing, we observed mid-term nuclear cells and analyzed the karyotype by the microscope.

### Characterization of McAbs

The heavy and light chain composition of 3 McAbs were determined using the ISO Strip Kit (Santa Cruz Biotechnology, Inc, USA).

The extent of McAbs binding to various normal and malignant cells (listed in Figure 1B) were determined with 0.1 mL of culture supernatant from positive hybridoma cells and 1×10^5^ target cells using ELISA for the screening described above, and the production of substrate was measured spectrophotometrically at 450 nm.

### Indirect Immunofluorescence Analysis

Indirect immunofluorescence was performed as follows. Briefly, cells were grown to 80% confluence on cover slips and then fixed in 95% ethanol at room temperature. After washing with PBS, the cell membranes were broken in triton X-100 for 20 min and blocked with 5% BSA for 60 min at room temperature, and then incubated with purified NJ001 (1∶80) at 37°C for 1 h. This was followed by incubation with FITC conjugated goat-anti-mouse IgG (1∶100) for 45 min at room temperature. The slides were counterstained with Hoechest. Immunofluorescence staining results were obtained using fluorescence and confocal microscopy (Zeiss LSM 710, Germany).

### Western Blot Analysis

In order to perform the Western blot analysis, the nine cell lines were initially cracked by the RIPA lysis buffer (50 mM Tris-HCl, 1% NP-40, 0.25% Na-deoxycholate, 50 mM NaCl, 1 mM EDTA, 1 mM PMSF, 1 mg/mL Aprotinin, 1 mM Na_3_VO_4_, 1 mM NaF). Next, 25 µg of total protein from the nine cell lines was electrophoresed on a 12% SDS-PAGE gel, and then transferred to a PVDF membrane (Amersham Pharmacia Life Science, USA). After blocking with 10% non-fat milk in TBST, the PVDF membrane was incubated with NJ001 (1∶300) and a anti-GAPDH antibody (Zhongshan Biological, Beijing, China) over night at 4°C to confirm equivalent protein loading in each lane. This was followed by incubation with HRP-conjugated goat-anti-mouse IgG for 1 h at room temperature. The PVDF membrane was further washed 3 times with TBST for 15 min each, and finally developed with ECL (Amersham Life Science) on X-ray film.

### Immunohistochemistry

NSCLC tissues (n = 106), SCLC tissues (n = 21), breast carcinoma tissues (n = 21), gastric cancer tissues (n = 5), colon cancer tissues (n = 5), ovarian cancer tissues (n = 5), liver cancer tissues (n = 5), pulmonary pseudotumor tissues (n = 25) and adjacent nontumourous lung tissues (n = 8) were obtained from the department of pathology in the first affiliated hospital of Nanjing Medical University. NSCLC tissues included lung adenocarcinoma tissues (n = 58) and squamous lung cancer tissues (n = 48). Tissue sections were treated with 0.3% hydrogen peroxidase for 5 min, followed by 30 min blocking with normal goat serum at room temperature. NM001 (1∶200) was applied to the blocked sections and incubated overnight at 4°C. The sections were incubated for 30 min at 37°C with HRP-labeled goat-anti-mouse IgG antibody (1∶2,000), and the positive signals were visualized by development in diaminobenzidine tetrahydrochloride (DAB) solution. The sections were viewed under an Olympus Ax-70 DMC Ie CCD camera connected to a PC monitor.

### Cell Proliferation Assay

SPC-A1 cells were seeded in a 96-well plate at 5,000 cells per well. Culture supernatants from positive hybridoma cells and SP2/0 cells were added. During the final 16 h of the 24 h, 48 h, and 72 h incubation at 37°C, the cells were pulsed with 0.5 µCi/well [^3^H] thymidine. Proliferation assays were performed by liquid scintillation counting of the harvested cells. Results of SPC-A1 cell proliferation measurement were presented as the count per minute (cpm).

### Soft Agar Assay

Colony formation was analyzed by soft agar assay. using the anchorage-dependent, lung adenocarcinoma cell line SPC-A1 as a tumor cell model, according to the procedures of Hong KW [40]. Briefly, a bottom layer of 0.5% agarose (Promega, U.S.A) containing of 2 mL culture medium was initially solidified in a 6-well culture plate. Next, 2 mL of 0.3% agarose solution containing 2×10^4^ cells with different concentrations of NJ001 or MCA2849 (irrelevant McAb) (0, 100, 200, 400, 800, or 1000 µg/mL) was layered on top. Each dose was tested in triplicate. After incubation at 37°C with 5% CO_2_ atmosphere for 2 weeks, the colonies that contained over 50 cells were counted under a microscope. The colony formation efficiency and the inhibition ratio of the colony were calculated as the following formulas: colony formation efficiency = (average number of colonies/average number of cells added per well)×100%; inhibition ratio of colony formation = (1- average number of colonies in NJ001 or MCA2849 group/average number of colonies in McAb free group)×100%. This experiment was repeated 3 times.

Anti-tetanus McAb (MCA2849) (AbD Serotec, Germany) was used as irrelevant McAb in this study.

### Xenograft Experiment

Thirty female BALB/c nude mice of 6-week old were purchased from Shanghai SLAC Laboratory Animal Co. Ltd. (Shanghai, China). The SPC-A1 cells were maintained in 10% FBS RPMI1640 medium until the cells reached 80% confluence. All procedures were conducted in accordance to the Animal Care and Use Committee guidelines of Nanjing Medical University.

In the preliminary study, SPC-A1 cells were incubated with NJ001 at 37°C in a 5% CO_2_ incubator for 2 h. Thus each 200 µL saline contained 2×10^6^ SPC-A1 cells and 400 µg NJ001. The lateral axilla of 5 nude mice were inoculated subcutaneously with the solution, and the control group, also composed of 5 mice, was injected the equivalent SPC-A1 cells in the same position. After three weeks of inoculation, mice were euthanized and the tissues were obtained for H&E staining.

The mice were randomly divided into 4 groups, with 5 mice per group. The xenograft was established by subcutaneous injection of 2×10^6^ SPC-A1 cells/200 µL per mouse into the lateral axilla. Antibodies were administered intraperitoneally at 3 different doses (200 µg, 400 µg, or 800 µg per mouse). Treatment was initiated simultaneously with the implantation and consisted of two steps: daily injection for the first week, followed by injections twice a week for the proceeding two weeks. The control group received sterile saline injections in the same mode. Animals were monitored for tumor size at 4-day intervals. The tumor volume (mm^3^) was calculated according to the following equation: Volume = width^2^×length/2 [28], [41]. All mice were euthanized three weeks after the initiation of treatment and tumors were removed and weighed. Tumor growth inhibition was calculated by the formula: tumor growth inhibition ratio = (1−average tumor weight in NJ001 group/average tumor weight in control group)×100%. Treatment toxicity was assessed by the physical appearance of the animals.

### H&E Staining

Tissues obtained were fixed in 10% formalin and embedded in paraffin. The embedded tissues were subsequently cut into 4 µm sections and placed on glass slides for H&E staining.

### Apoptosis Assay

SPC-A1 cells were seeded in a 12-well plate at 1×10^5^ cells per well. After overnight incubation, SPC-A1 cells were cultured with or without 200 µg/mL NJ001 or MCA2849 for 24 h and 48 h. Each time point was tested in triplicate. The morphological changes of the cells were then observed and the rate of apoptosis was determined by flow cytometry. Briefly, cells were collected, washed with PBS and resuspended in 500 µL binding buffer containing 10 mmol/L HEPES-NaOH (pH 7.4), 140 mmol/L NaCl, and 2.5 mmol/L CaCl_2_. Next, 5 µL of Annexin V-FITC (Bender MedSystems, Austria) and 5 µL of propidium iodide (PI) solution (Bender) were added and the cells were then incubated in the dark for 15 min. The fluorescence was then analyzed by flow cytometry. Early apoptosis and late apoptosis was determined as the percentage of Annexin V^+^/PI^−^ cells and Annexin V^+^/PI^+^ cells, respectively. The rate of total apoptosis was the sum of early and late apoptosis. This experiment was repeated 3 times.

### Statistical Analysis

All values were expressed as mean ± standard deviation. Mean comparison of groups was conducted using single factor variance analysis. The pairwise comparison was performed with the LSD test if the variance was homogeneous and a P-value<0.05 was regarded as statistically significant, while Dunnett's C method was used for the heterogeneity of variance, setting 0.05 as the significance level.
